# Traditional Chinese medicine for adhesive intestinal obstruction: theory, methods and mechanisms of action

**DOI:** 10.3389/fmed.2025.1573655

**Published:** 2025-05-02

**Authors:** Pengfei Zhou, Huiju Yang, Jiawen Wang, Mingming Sun, Shuai Yan

**Affiliations:** ^1^Department of Anorectal Surgery, Longhua Hospital, Shanghai University of Traditional Chinese Medicine, Shanghai, China; ^2^Department of Aolorectal Surgery, The Third Affiliated Hospital of Henan University of Chinese Medicine, Zhengzhou, China; ^3^Department of Anorectal Surgery, Suzhou TCM Hospital Affiliated to Nanjing University of Chinese Medicine, Suzhou, China

**Keywords:** adhesive intestinal obstruction, traditional Chinese medicine, Chinese materia medica, acupuncture, mechanism

## Abstract

Adhesive intestinal obstruction (AIO) represents a common postoperative complication, particularly following abdominal surgery, with reported incidence rates varying between 50 and 80%. Traditional Chinese Medicine (TCM) has proven clinically effective in managing AIO, offering diverse therapeutic approaches that facilitate multi-pathway and multi-target treatment. Clinical evidence consistently supports the favorable safety profile of Traditional Chinese Medicine (TCM). Nevertheless, several critical challenges remain to be addressed, including its complex multi-component nature, insufficiently elucidated mechanisms of action, lack of precise dosage standards, inconsistencies in decoction preparation methods, inconvenient administration procedures, and a paucity of large-scale, multicenter clinical trials with robust evidence. These barriers hinder the widespread adoption and clinical integration of TCM. Moving forward, large-scale, multicenter studies are essential to further investigate the safety and efficacy of TCM. Moreover, reforms in its administration methods and deeper exploration of its mechanisms in AIO treatment are crucial.

## Introduction

Adhesive intestinal obstruction (AIO), a common surgical condition caused by intestinal or intra-abdominal adhesions, develops in an estimated 70–90% of patients following abdominal surgery (1). Dembrowski first reported data on the induction of adhesions in animal models in 1889, and since then, numerous *in vitro* and *in vivo* studies have explored this phenomenon (2). Abdominal adhesions primarily occur in the postoperative period following surgeries on the intestines, colon, appendix, and uterus. These adhesions typically manifest as pathological bands between the greater omentum, large intestine, abdominal wall, and other intra-abdominal organs. These bands may consist of connective tissue membranes, thick fibrous bridges containing vascular nerve tissue, or direct adhesions between two organ surfaces. While most adhesions are asymptomatic, scar tissue is particularly prone to causing intestinal obstruction due to its inelasticity (3).

Current treatment options for intestinal obstruction encompass both surgical and non-surgical approaches. Surgical treatment allows precise identification and management of the adhesion site but is invasive, carries the risk of forming new adhesions, and may necessitate repeat surgeries, leading to recurring AIO. Laparoscopic treatment, while minimally invasive and gentler on intestinal tissues, does not guarantee that adhesions will not recur. Non-surgical treatments, such as water fasting, gastrointestinal decompression, anti-infective drugs, acid–base balance maintenance, and electrolyte balance support, offer symptomatic relief. Medications like octreotide and pantethine are commonly used, although they may not always prove effective. Despite advancements in surgical techniques aimed at reducing adhesion formation, it remains an unavoidable risk.

In recent years, Traditional Chinese Medicine (TCM) has been increasingly used by Chinese physicians for treating AIO. TCM therapies, including oral administration, enemas, acupuncture, and naprapathy, have shown promising clinical results. This study reviews the pathogenesis and treatment of AIO, highlighting the strengths and limitations of representative TCM therapies, with the aim of providing insights for clinical management.

## Search strategy

**Figure 1:**
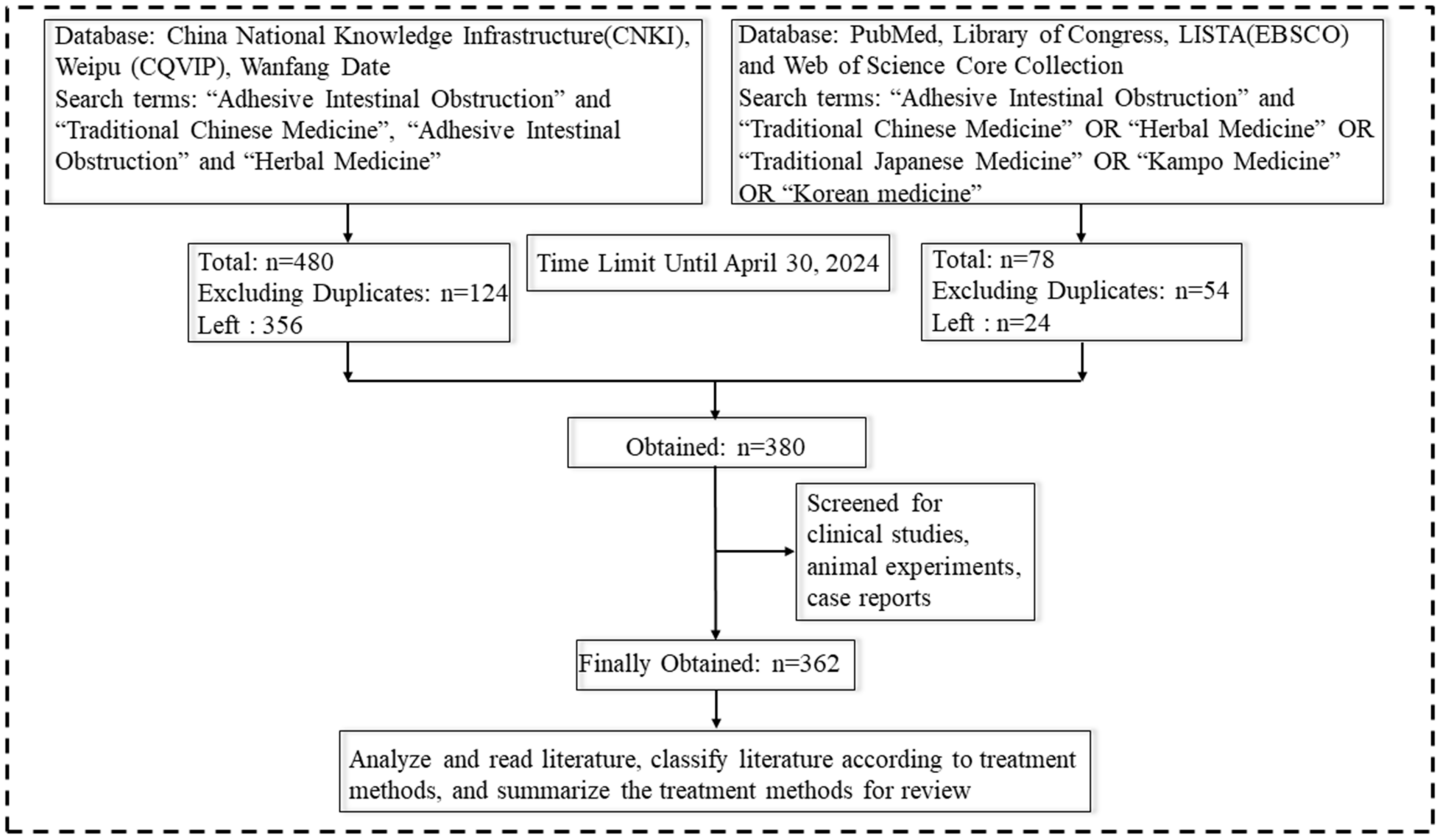
Literature retrieval strategy. Created with BioGDP.com (4).

The search terms “Adhesive Intestinal Obstruction” and “Traditional Chinese Medicine,” “Adhesive Intestinal Obstruction” and “Herbal Medicine,” “Traditional Japanese Medicine” and “Adhesive Intestinal Obstruction,” “Kampo Medicine” and “Adhesive Intestinal Obstruction,” and “Korean medicine” and “Adhesive Intestinal Obstruction” were used in both Chinese and English. Literature searches were conducted in the China National Knowledge Infrastructure (CNKI), Weipu (CQVIP), Wanfang Data, PubMed, Library of Congress, LISTA (EBSCO), and Web of Science Core Collection until April 30, 2024. A total of 527 Chinese articles were retrieved, with 356 remaining after excluding duplicates. In addition, 78 English articles were obtained, with 24 left after excluding duplicates. After screening for clinical studies, animal experiments, and case reports, 380 articles were initially reviewed. Upon further analysis and categorization based on treatment modality, 362 articles were finally included for summarization in this review, as shown in Figure 1.

## Epidemiology

Intestinal obstruction represents a frequent surgical presentation, with adhesions constituting the predominant etiology. Clinical data indicate that approximately 20% of cases manifest as acute abdominal pain, while 80% involve small bowel obstruction. In contrast, large bowel obstruction most commonly results from colorectal carcinoma. The cardinal clinical manifestations comprise abdominal pain, vomiting, abdominal distension, and absolute constipation. Adhesive small intestinal obstruction can be classified as congenital or acquired. Congenital forms, though uncommon in normally developed pediatric patients, typically arise from developmental anomalies including intestinal dysgenesis or secondary to meconium peritonitis. Acquired small intestinal obstruction is far more prevalent, typically following abdominal surgery. Postoperative adhesions develop in nearly all patients undergoing laparotomy (5), with peritoneal adhesions affecting 93% of patients after cesarean section (6). The incidence of peritoneal adhesions following laparoscopic surgery is slightly lower, approximately 45% (7). In patients undergoing open lower abdominal surgery, adhesions occur in 67–93% of cases, but only 5–18% experience symptoms of intestinal obstruction, with complication rates varying based on surgical type and postoperative follow-up duration (8). Post-cesarean small bowel obstruction occurs in approximately 1% of patients within the first postoperative year, with the cumulative incidence rising to 3% during long-term follow-up (9). Postoperative abdominal adhesions account for 70% of intestinal obstruction cases requiring hospitalization. They are associated with approximately 3% of cesarean sections and 1% of all surgical admissions (10). Among surgical interventions, total rectocolectomy (15%), total colectomy (8%), and ileostomy (10%) carry the highest readmission risks attributable to postoperative adhesions. Beyond surgical complications, various inflammatory conditions may also precipitate adhesive intestinal obstruction (AIO), including pelvic inflammatory disease, gastroenteritis, appendicitis, Crohn’s disease, ulcerative colitis, as well as traumatic injuries (11). Additional risk factors for AIO include peritonitis, greater omentectomy, and penetrating abdominal trauma (12). Surgeries involving the upper abdomen, including the stomach, gallbladder, or pancreas, carry a lower risk of adhesion formation. Furthermore, age negatively correlates with adhesion development, with patients under 60 years old being at the highest risk. Surgery involving the colon for peritonitis and colon cancer is also associated with an increased risk of adhesion-related complications (13). Postoperative adhesions occur in 60–90% of women following gynecologic surgery and represent a significant contributing factor to secondary infertility (14). The incidence of postoperative adhesions is comparable between gynecologic cesarean and laparoscopic surgeries, with similar readmission rates attributed to adhesion-related complications (15).

## Pathogenesis

AIO can be classified into high small intestinal obstruction, low small intestinal obstruction, and colonic obstruction based on the site of involvement. Adhesion formation commonly occurs following abdominal surgery as a natural component of the wound-healing response. The extent of adhesion formation depends on the nature of the surgery and individual patient responses. Peritoneal adhesions represent pathological fibrous connections between adjacent anatomical structures. These abnormal attachments exhibit diverse morphological characteristics, from delicate avascular membranes to dense vascularized fibrous bands incorporating neural elements, or even complete fusion of organ surfaces. Their pathogenesis originates from an inflammatory cascade initiated by tissue trauma. During healing, this inflammation can affect surrounding normal tissues and organs (16). Adhesion formation starts following peritoneal injury, initiating a cascade of inflammatory responses. Surgical trauma, such as abrasion, disrupts the peritoneal mesothelium, releasing fibronectin and other inflammatory mediators, including leukocytes and mesothelial cells. During the healing process, fibrin deposition occurs on the injured mesothelial surfaces, forming provisional connective bridges between opposing tissues. Locally secreted fibrinolytic factors may partially or completely resolve these fibrin matrices. Factors such as surgery, infection, and hypoxia reduce fibrinolytic activity, allowing fibroblasts and other cells to migrate through the remnants of the fibrin bridges, which eventually develop into adhesions (17). In some cases, adhesions lead to further obstruction, with over 70% of small bowel obstructions being secondary to adhesions (18). The key pathological processes in postoperative abdominal adhesions are the inflammatory response and collagen deposition. While an appropriate inflammatory response facilitates tissue repair, excessive inflammation indirectly promotes collagen deposition, leading to abdominal adhesion formation (19). C-X-C motif chemokine ligand 2 (CXCL2), also known as macrophage inflammatory protein (MIP)-2, has been shown to be closely linked to postoperative abdominal adhesions (20). Studies have indicated that certain components of Chinese medical can inhibit the CXCL2-CXCR2 pathway, reducing inflammation and collagen deposition, thus preventing the formation of postoperative abdominal adhesions (21).

**Figure 2:**
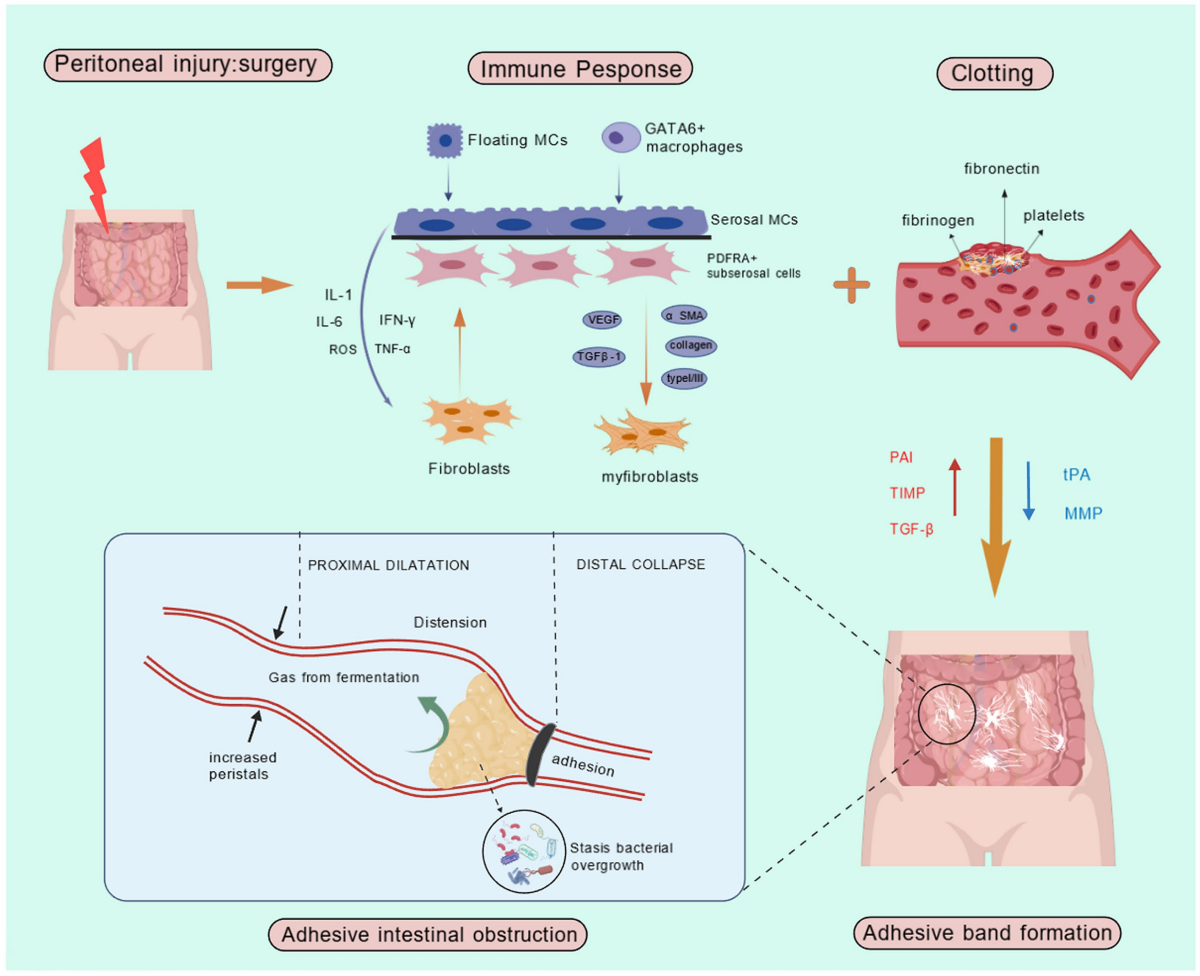
Pathogenesis of AIO. IL-1, Interleukin-1; IL-6, Interleukin-6; ROS, Reactive Oxygen Species; IFN-γ, Interferon-gamma; TNF-α, Tumor Necrosis Factor-alpha; MCs, Mast Cells; PAI, Plasminogen Activator Inhibitor; TIMP, Tissue Inhibitor of Metalloproteinases; TGF-β, Transforming growth factor-beta; tPA, Tissue-type plasminogen activator; MMP, Matrix Metalloproteinase.

The most common site for adhesion formation is between the greater omentum and the midline closure (16). Peritoneal tissue repair is a complex process involving various types of cells, cytokines, coagulation factors, and proteases that cooperate to restore tissue integrity (22). Inflammation, angiogenesis, and other factors regulate adhesion formation during tissue repair (22). Adhesion formation arises from an imbalance between fibrin deposition and degradation, a critical determinant of postoperative peritoneal healing. Surgical injury triggers bleeding, increased vascular permeability, and extravasation of fibrinogen-rich fluid. Thrombin then mediates fibrinogen-to-fibrin conversion, culminating in the intraperitoneal coagulation cascade (23). When systemic coagulation is impaired, fibrin deposition occurs, forming the foundation for fibrocollagenous tissue development and extracellular matrix formation. Simultaneously, the inflammatory response begins, characterized by the migration of inflammatory cells, cytokine release, and coagulation cascade activation. This activation leads to the formation of thrombin, which facilitates the conversion of fibrinogen to fibrin (24). The activation of the fibrinolytic system within the first 5–7 days after peritoneal injury is critical for preventing adhesion formation. Fibrin degradation into split products is predominantly triggered by fibrinogen’s conversion to fibrinolytic enzymes. The two key physiological activators, tissue-type plasminogen activator (tPA) and urokinase-type plasminogen activator (uPA), are synthesized and stored in endothelial cells, mesothelial cells, and macrophages. tPA, a serine protease, exhibits the highest fibrinogen-activating efficacy due to its strong binding affinity for fibronectin. This activation process is particularly amplified at anatomical sites susceptible to adhesion formation. tPA mediates approximately 95% of fibrinogen activation within the abdominal cavity, whereas uPA contributes to fibrin matrix degradation through distinct mechanisms (25). While uPA exhibits lower fibrin affinity and consequently reduced fibrinogen activation compared to tPA, it plays a more prominent role in tissue remodeling and alternative fibrinolysis pathways. Inflammatory mediators, including transforming growth factor beta and interleukins, play a central role in adhesion formation. These mediators decrease the fibrinolytic capacity of the peritoneum, thereby increasing adhesion formation. Fibronectin aids tissue repair by restoring and depositing along surfaces. Fibrin, a sticky substance, facilitates the adhesion of organs or the fusion of injured plasma surfaces. Although the fibrin matrix deposited during wound healing is transient, locally released fibrinolytic enzymes degrade these provisional adhesions within 72 h post-injury. This highlights fibrinolysis’ dual role extending beyond vascular thrombus resolution to encompass tissue remodeling. Through fibrin cleavage, mesothelial cell proliferation is facilitated, enabling complete peritoneal re-epithelialization within 4–5 days and preventing permanent adhesion formation (26). Adequate blood flow is essential for fibrinolysis, and ischemia resulting from peritoneal injury disrupts this process. During tissue repair, the provisional fibrin matrix undergoes progressive organization through infiltration of collagen-producing fibroblasts and other reparative cells. Concurrently, fibrinolytic activity is modulated by endogenous protease inhibitors (e.g., α1-antitrypsin and α2-antiplasmin), although their specific regulatory roles in peritoneal fibrinolysis require further elucidation (27). The equilibrium between fibrinogen activators and inhibitors critically regulates peritoneal tissue repair, determining whether normal healing or pathological adhesion formation occurs. When fibrinolytic activity is insufficient, the provisional fibrin matrix fails to undergo timely degradation and instead undergoes fibroblastic transformation. Infiltrating fibroblasts deposit collagenous extracellular matrix while secreting angiogenic factors that induce neovascularization. Ultimately, these vascularized fibrotic tissues become enveloped by peritoneal mesothelium, culminating in the establishment of permanent adhesions (Figure 2) (26).

Beyond the inflammatory cascade, intestinal flora may contribute to adhesion formation. Specifically, colonization of the intestinal flora is associated with a four-fold reduction in the risk of peritoneal and peri-anastomotic adhesion formation (28). The inflammatory action of bacterial translocation is mediated through the activation of cell-mediated immune responses, particularly macrophages and the expression of interleukin-6 (29). This process can ultimately lead to the formation of peritoneal adhesions (30). Reducing the intestinal flora load may impact the incidence of postoperative adhesions, as animal studies have shown that lowering the number of intestinal flora decreases the formation of experimental intra-abdominal adhesions (31).

## Diagnostic measures

In clinical practice, imaging techniques such as conventional radiology, ultrasound, computed tomography (CT), and magnetic resonance imaging (MRI) are frequently used to diagnose intestinal obstruction, determine its location, assess the pathogenesis, and evaluate the severity of the obstruction (32). The most commonly used adjunctive measures include supine and upright abdominal radiographs. Abdominal X-rays are advantageous for their rapid and cost-effective nature, with an accuracy of approximately 64–83% in diagnosing adhesive small intestinal obstruction (33). Radiographic signs of intestinal obstruction often include intestinal collaterals greater than 3 cm in diameter, multiple air-fluid levels, and the absence of air in the colon (34). Typical imaging findings can support the diagnosis of AIO, with CT scans offering high diagnostic value (35). CT scans are particularly useful for determining the extent of the obstruction and distinguishing whether it is caused by adhesive bands or adhesions (36). Abdominal imaging can help identify the gas and fluid planes of intestinal obstruction, locate the obstruction, and uncover its underlying cause. The diagnostic accuracy of CT for adhesive small intestinal obstruction is approximately 95% (37). In patients with intestinal obstruction, CT imaging typically reveals features such as intestinal dilatation, gas and fluid accumulation, inhomogeneity of gas and fluid planes, and a high column of gas in the intestinal tract. Additional signs such as aggregation of intestinal collaterals, the bird’s beak sign, the fat notch sign, and the small-bowel feces sign are commonly associated with adhesive small bowel obstruction (36). CT imaging is valuable for assessing whether surgical intervention is necessary and can also detect localized intestinal wall ischemia (38). However, imaging methods like ultrasound and CT are challenging to interpret, and there remains a risk of misdiagnosis or underdiagnosis in clinical practice (39). Ultrasound diagnosis is highly operator-dependent, and air within the intestine can obscure the condition. Moreover, diagnosing obese patients is particularly challenging using ultrasound. Water-soluble contrast agents, such as meglumine amidotrizoate (gastrografin), are beneficial for diagnosing patients undergoing non-surgical treatments. These agents not only serve diagnostic purposes but also have therapeutic value by promoting water entry into the small bowel lumen, reducing intestinal wall edema, and enhancing smooth muscle contractility to induce peristalsis and relieve obstruction symptoms (40). Small intestinal enemas are useful for diagnosing low-grade or partially intermittent small intestinal obstruction. Approximately 200–250 mL of barium, followed by 1–2 L of methylcellulose solution, is injected into the proximal jejunum through a long nasoenteric catheter. Fluoroscopic observation of motility then provides valuable information about the obstruction’s location, extent, and severity, aiding diagnosis (41). However, a drawback of this method is that the contrast agent moves slowly through the fluid-filled, hypotonic small intestine.

## Modern medical therapeutic measures in AIO

The treatment of AIO should be tailored to the patient’s clinical symptoms. While surgical treatment can effectively address the obstruction, it may also lead to the formation of new adhesions, whereas conservative treatment does not eliminate the underlying cause of the obstruction (42). Non-surgical treatment is appropriate in the absence of peritonitis or strangulation signs, but it is associated with a high recurrence rate and short intervals between readmissions (43). In cases of conservative management, surgery is recommended if the intestinal obstruction persists for more than 3 days or if drainage exceeds 500 mL on day 3 (44). Surgery should also be considered if the patient develops fever or elevated white blood cell counts during treatment (45). Postoperative peritoneal adhesions are a common consequence of surgery, for which effective preventive measures have yet to be identified. Although meticulous surgical techniques and physical barriers can help prevent adhesions, therapeutic strategies capable of preventing or reducing adhesion formation require further investigation (46). The goal of non-surgical treatment for AIO is to enhance gastrointestinal motility, facilitating the passage of intestinal contents, and thereby alleviating symptoms such as abdominal pain and bloating. Key therapeutic measures include gastrointestinal decompression, antispasmodic and analgesic agents, antibiotic use, maintenance of fluid and electrolyte balance, and parenteral nutritional support. Despite promising results in animal studies, drugs such as non-steroidal anti-inflammatory drugs, corticosteroids, calcium channel blockers, antihistamines, and colchicine have shown disappointing outcomes in human trials (27).

### Catheterization of intestinal obstruction

Adjunctive therapy, such as intestinal obstruction catheterization, can complement conservative treatment by maintaining electrolyte balance, providing nutritional support, and preventing infection. Conventional nasogastric decompression tubes, which are typically short, are limited to draining fluids from the stomach and cannot reach the small intestine to remove trapped gasses and fluids. The intestinal obstruction catheter offers a superior solution, as it is guided through the nasal cavity, pylorus, and duodenum to the upper jejunum. The catheter’s negative pressure drainage significantly improves gastrointestinal decompression compared to conventional methods (47).

### Surgical treatment

Surgery remains necessary in some cases, though it may lead to new adhesions, with 10–30% of recurrent intestinal obstructions requiring repeat abdominal surgery (27). Open abdominal surgery remains the standard treatment for adhesive small bowel obstruction. However, laparoscopic intervention—which minimizes peritoneal trauma through reduced tissue handling—may offer the additional advantage of lowering adhesion formation (48). Successful laparoscopic management is contingent upon several key factors, including hemodynamic stability, the absence of peritonitis or severe intra-abdominal sepsis, and the surgeon’s technical proficiency and experience (49). Laparoscopic adhesiolysis confers multiple clinical benefits compared to open procedures, notably diminished postoperative pain, accelerated return of bowel function, reduced hospitalization duration, and enhanced overall recovery (50).

### Antifibrotic drugs

*Citric acid* and *heparin* reduce fibrin deposition (14), while *halofuginone*, an inhibitor of type I collagen, prevents adhesion formation by reducing collagen deposition in the fibrin matrix (51). Despite their proven efficacy in animal models, further clinical studies are needed to determine their applicability in human medicine.

### Antibacterial

Cyclooxygenase (COX) inhibitors, both non-selective (e.g., *indomethacin* and *nimesulide*) and selective (e.g., *celecoxib* and *rofecoxib*), have demonstrated effectiveness in reducing the initial inflammatory response, vascular permeability, and inflammatory factors in mouse models (52). Although these drugs show promise in preclinical studies, their effectiveness in preventing adhesion formation requires further investigation.

### Fibrinolytic agent

Fibrinolytic agents such as *streptokinase* and *urokinase* promote fibrinolysis by enzymatic degradation of fibrin. However, their clinical use is limited due to high costs and an increased risk of bleeding (23).

### Lubricants

Lubricants, which are glycoproteins present in articular cartilage and known for their anti-adhesive properties, have shown efficacy in reducing abdominal adhesions in rat models with minimal side effects. However, human studies on their effectiveness are still lacking (53).

### Barrier agent

The use of physical barriers has also been explored as a preventive measure against adhesions. Solid or liquid physical barriers can cover damaged peritoneal tissue, keeping it separated until reepithelialization is complete (54). Modified oxidized regenerated cellulose (Interceed) has been shown to be effective in humans, forming a gel that physically separates tissue layers during the healing process. Bioresorbable membranes, such as *hyaluronan* and *carboxymethyl cellulose-based membranes* (e.g., *Seprafilm*), serve as barriers, maintaining intestinal separation until the mesothelial lining is restored (24). Icodextrin 4% (Adept), approved by the *US Food and Drug Administration (FDA)*, is injected into the abdomen post-surgery to separate damaged peritoneal surfaces from other structures during the early postoperative period. *SprayGel*, a powdered gel that lasts for 5–7 days before being absorbed, provides a more viscous solution compared to Adept, offering a more effective barrier to adhesion formation. With intensive research into the mechanisms of adhesion development, various barrier materials such as *hyaluronic acid, carboxymethyl chitin, and oxidized regenerated cellulose* have been successfully used in clinical practice to prevent adhesions by separating damaged tissues and promoting mesothelial cell repair (55).

## The strategy of TCM or TCM combined with modern medical therapeutic measures to treat AIO

TCM offers a wide array of therapeutic approaches, often incorporating multiple modalities. Fundamentally, TCM is patient-centered, focusing on both symptom relief and addressing the root causes of ailments. Treatments encompass oral medications, enemas, acupuncture, cupping, and the topical application of traditional herbal remedies. Central to TCM are theories such as *qi* and *blood* theory, where *qi* represents the intangible, high-mobility substance essential for life activities, while *blood* refers to the red liquid circulating through the vessels to nourish and hydrate the body. Additionally, the *Zang–Xiang* theory links internal organs (*Zang*) with their outward manifestations (*Xiang*), including physiological functions and pathological changes. TCM treatment strategies are individualized based on the patient’s specific condition, with common formulas like *Major Purgative Decoction*—comprising *Rhubarb* (12 g), *Aurantii Fructus Immaturus* (12 g), Magnoliae Officinalis Cortex (24 g), and *Natrii Sulfas* (9 g)—often employed. Other widely used formulas include *Peach Kernel Qi-Guiding Decoction*, which contains *Persicae Semen* (12 g), *Rhubarb* (12 g), *Cinnamomi Ramulus* (6 g), *Natrii Sulfas* (6 g), and *Glycyrrhizae Radix et Rhizoma* (6 g), and *Blood* S*tasis-Expelling Decoction*, consisting of *Persicae Semen* (12 g), *Carthami Flos* (9 g), *Angelicae Sinensis Radix* (9 g), *Rehmanniae Radix* (9 g), *Chuanxiong Rhizoma* (4.5 g), *Paeoniae Radix Rubra* (6 g), *Achyranthis Bidentatae Radix* (9 g), *Platycodonis Radix* (4.5 g), *Bupleuri Radix* (3 g), *Aurantii Fructus* (6 g), and *Glycyrrhizae Radix et Rhizoma* (6 g). These formulas, among others, are routinely employed in the treatment of AIO in TCM. In addition to the classic formulas widely used in TCM, practitioners frequently employ customized combinations of herbs tailored to the patient’s symptoms, aiming to improve therapeutic outcomes and alleviate discomfort. Clinically, treatments for AIO in TCM encompass various approaches, including herbal medicinal liquid enemas, gastric lavage, acupuncture, topical applications, and other methods. Through these diverse techniques, TCM effectively mitigates AIO symptoms, with the ultimate goal of achieving a cure. Integrating TCM with modern medical practices has demonstrated improved treatment efficacy, often combining oral or enema administration of herbal remedies, external herbal patches, acupuncture, and acupoint therapy alongside modern life support measures. This integrative approach offers significant advantages, particularly in alleviating AIO symptoms and reducing the need for surgical intervention. In most clinical settings, AIO treatment begins with gastrointestinal decompression and basic life support, followed by TCM interventions such as enemas, gastric lavage, or acupoint injections. However, the optimal timing and conditions for TCM intervention remain unclear, necessitating further research to determine the most effective stages for TCM application in AIO treatment. TCM prioritizes preventive care before disease onset, and understanding how to effectively apply TCM to prevent the progression of AIO is a critical area for further investigation. The most commonly used treatment modalities are summarized in Table 1.

**Table 1:** Methods and advantages of traditional Chinese medicine in the treatment of adhesive intestinal obstruction.

Name	Methods	Examples of treatments	Function	Advantages	Effective rate
Decoction of TCM	Gavage or enema	*Major Purgative Decoction* is administered to the patient either via a nasogastric tube, where it is introduced into the stomach, or through an enema, where it is delivered into the rectum (96)	Regulating the intestines and alleviating diarrhea caused by heat evil (a pathogenic factor in TCM)	TCM formula *Major Purgative Decoction* can enhance clinical efficacy by promoting bowel movements, alleviating constipation, and facilitating the patency of intestinal obstructions	96.5%
Acupoint directional drug delivery	Chinese materia medica are ground into powder to be applied to the electrode pads and affixed to the acupoints for treatment	Grind 12 g of *Rhubarb root*, 12 g of *Aurantii Fructus Immaturus*, 24 g of *Magnoliae Officinalis Cortex*, and 9 g of *Natrii Sulfas* into a fine powder. Mix with vinegar to form a paste, then apply it to the following TCM acupoints: Zhongwan (CV12), Shenque (CV8), Qihai (CV6), Zusanli (ST36), and Xiajuxu (ST39) (97)	The drug’s effects are exerted through the acupoints, enabling both transdermal absorption and stimulation of meridian acupuncture points	The powdered Chinese medicine, activated with vinegar, utilizes intermediate frequency current therapy to enhance drug absorption	87.18%
External application of TCM around the umbilicus	Crude drugs are placed in an external bag and applied externally around the patient’s umbilicus	Crush 100 g of *Rhubarb* root, 200 g of *Natrii Sulfas*, and 80 g of *Borneolum Syntheticum*, then package them in a disposable non-woven external bag. When applied, place the bag flat around the patient’s umbilicus (46)	The medicines enter the bloodstream through the superficial veins of the anterolateral abdominal wall at the umbilicus, thereby exerting their therapeutic effects	Significant reduction in the time to relief of abdominal distension, time to voluntary gas evacuation, and time to voluntary defecation. Additionally, serum neutrophil percentage and C-reactive protein (CRP) levels were markedly decreased	97.1%
TCM combined with *Meglumine Diatrizoate*	An herbal enema is administered along with 76% meglumine diatrizoate injected into the stomach, followed by clamping the gastric tube	Gastrointestinal decompression and supplementation of water and electrolytes were administered to maintain the patient’s acid–base balance. 100 mL of 76% *meglumine diatrizoate* was injected into the stomach, followed by clamping of the gastric tube. *Major Purgative Decoction*, prepared with 500 mL of water, was administered as an enema twice daily for three consecutive days of treatment (98)	*Meglumine diatrizoate* helps dilute intestinal contents and expand the distal obstruction site, while the Chinese medicine exerts its effects through absorption via the intestinal mucosa	Improvements in the time to relief of abdominal pain, time to anal defecation, time to stool drainage, time to disappearance of abdominal pressure, and time to recovery of bowel sounds	93.88%
TCM combined with Zusanli (ST 36) acupoint injection	Gastric lavage with TCM is performed, followed by the injection of vitamin B1 or neostigmine at the Zusanli acupoint	*Compound Major Purgative Decoction*, a TCM formula consisting of 15 g of *Magnoliae Officinalis Cortex*, 6 g of Aurantii *Fructus*, 10 g of *Paeoniae Radix Rubra*, 15 g of *Raphani Semen*, 10 g of *Aucklandiae Radix*, 10 g of *Albizia julibrissin Durazz*, and 10 g of *Sennae Folium*, is decocted in 500 mL of water. It is administered orally or injected into the gastric tube in two divided doses. Additionally, 60 mg of vitamin B1 is injected into Zusanli (99), or 1 mg of neostigmine is given via bilateral Zusanli injections (100)	Chinese medicines improve intestinal motility and blood flow, enhance capillary permeability, and possess antimicrobial properties, while promoting intra-abdominal blood absorption. Injections at the Zusanli acupoint help restore gastrointestinal peristalsis	Effectively alleviate symptoms such as gas and bowel movement issues, abdominal distension, and abdominal pain in the patient	85.7%
Enema of TCM combined with subcutaneous injection of octreotide acetate injection	Chinese Medicine Liquid is instilled into the stomach, while octreotide acetate injection is administered subcutaneously	In combination with basic nutritional support therapy, *Major Purgative Decoction* was administered via the gastric tube, along with subcutaneous injections of *Octreotide Acetate Injection* at a dose of 0.1 mg every 8 h (101)	TCM *Major Purgative Decoction* has anti-inflammatory effects, restores gastrointestinal motility, and promotes the clearance of obstructions	Promotes the resolution of gas, defecation, abdominal pain, bloating, and fluid accumulation while reducing the levels of inflammatory factors IL-6 and CRP	97.06%
Chinese Herbal enema combined with acupuncture	Chinese medicines are administered via injection into the anus, while acupuncture treatment is simultaneously performed	*Liqijieduhuoxue* Formula, a TCM formulation consisting of 25 g of *Angelicae Sinensis Radix*, 20 g of *Persicae Semen*, 15 g of *Cyperi Rhizoma*, 15 g of *Citri Reticulatae Pericarpium*, 15 g of *Aurantii Fructus*, 15 g of *Chuanxiong Rhizoma*, and 10 g of *Rhubarb*, is decocted with 200 mL of water, with one dose administered per day through the gastric tube. Acupuncture is performed at the TCM acupoints Tianshu (ST25), Zusanli (ST36), Zhongwan (CV12), Xiajuxu (ST39), and ShangJuxu (ST37), with each treatment lasting 30 min (102)	Chinese medicine help clear heat and remove toxins, activate blood circulation, and resolve blood stasis, while acupuncture provides bidirectional regulation of intestinal motility	Reduced the time to the first passage of gas and the time to the first bowel movement and decreased serum levels of inflammatory factors TNF-alpha and IL-6. Additionally, another meta-analysis demonstrated that the acupuncture group shortened hospitalization time and alleviated the clinical symptoms of AIO (103)	93.1%
TCM Hot Ampoule Combined with Electroacupuncture	A Chinese medicine hot compress pack is applied, combined with acupuncture at the Zusanli (ST36) and Zhigou (SJ6) acupoints	For external use, prepare a hot compress pack by mixing 250 g of salt and 250 g of *Euodiae Fructus* (Fruit), and processing with 30 mL of vinegar. Heat the mixture for 5–8 min until it reaches a temperature of approximately 45–50°C. Apply the hot compress pack to the Zhongwan (CV12) and Shenque (RN8) acupoints for 20–30 min each time. Simultaneously, provide acupuncture needle stimulation to the Zusanli (ST36) and Zhigou (SJ6) acupoints (104)	This therapy improves intestinal peristalsis and blood circulation while dredging the meridians and regulating gastrointestinal function	Effectively alleviates the patient’s symptoms, including abdominal pain, bloating, vomiting, difficulty with defecation, and poor gas evacuation	96.66%
Chinese the decoction of crude drugs with liquid paraffin therapy	Liquid paraffin and senna water by gastric tube	After 4 h of gastrointestinal decompression, with the gastric fluid volume falling below 50 mL per hour, 100 mL of liquid paraffin was injected through the gastric tube. Once the patient was free from vomiting and abdominal distension, 50 mL of Sennae Folium (leaf) water was then administered (105)	Senna water enhances the peristalsis of the patient’s gastrointestinal tract, while liquid paraffin lubricates the intestines without being absorbed	Significant relief of the patient’s clinical symptoms, with no recurrence observed during the 1–3 years of follow-up	92.6%
Chinese Medicine Acupuncture Point Patch Therapy	Grinding of Chinese medicine into fine powder for acupoint application	Acupoint application therapy involves a drug composition of 50 g of *Caryophylli Flos* (bud) and 50 g of *Cinnamomi Cortex* (bark), which is ground into a fine powder. The powder is mixed with vinegar to form a medicinal paste, which is then applied to the Shenque (RN8) acupoint at the umbilical region (106)	Chinese medicine applied to the umbilicus stimulates the nerves around the acupoints, activating the nervous, immune, and digestive systems	Combination of TCM acupoint application and stimulation of the Shenque acupoint demonstrates remarkable efficacy in the treatment of early inflammatory bowel obstruction	92.31%
TCM combined with catheterization of intestinal obstruction	TCM combined with catheterization of intestinal obstruction	Under the premise of fasting, gastrointestinal decompression, and complete parenteral nutritional support, a catheter for intestinal obstruction and nasoenteric tube administration were introduced. *Peach Kernel Qi-Guiding Decoction* was then administered through the nasoenteric tube (107)	Chinese medicine has the effects of activating blood circulation, removing blood stasis, and draining heat from the internal organs	*Peach Kernel Qi-Guiding Decoction*, combined with an intestinal obstruction catheter, significantly alleviates abdominal pain and distension, reduces levels of leukocytes, CRP, and tumor necrosis factor-α (TNF-α), and decreases the likelihood of requiring intermediate surgery	/
Chinese medicine hot pack combined with acupoint gua sha treatment	Chinese medicine hot pack combined with Zusanli, Yangchi, Zhigou, Dachangshu, Shenshu acupuncture points and peripheral scraping	The Chinese medicine formula, consisting of 120 g of *Psoraleae Fructus*, 150 g of *Raphani Semen*, 12 0 g of *Cuscutae Semen*, 120 g of *Perillae Fructus*, and 120 g of *Sinapis Semen*, is placed into a small cloth bag and heated to approximately 50°C. The bag is then applied to the patient’s navel. Scraping therapy is performed at the Zusanli (ST36), Yangchi (SJ4), Zhigou (SJ6), Dachangshu (BL25), and Shenshu (BL23) acupoints (108)	The hot herbal bag helps warm the middle jiao and promote the flow of *qi*. The scraping therapy, through local mechanical stimulation, facilitates the dredging of meridians and enhances the circulation of *qi* and *blood*	The therapy significantly improves the patient’s time to anal defecation and alleviates symptoms such as nausea and vomiting	96%
Hot compresses of Chinese medicine combined with warm moxibustion at acupoints	TCM Hot Compress Combined with Acupuncture Points and Ginger Moxibustion through Ginger	Soak a dry towel in warm water with a few drops of peppermint oil, wring out the excess water, and fold the towel over the abdomen below the umbilicus, ensuring it covers the Yinjiao (SP6), Qihai (CV6), Shimen (CV8), Guanyuan (RN4), and other acupoints. Simultaneously, apply ginger moxibustion treatment to the Shenque (RN8) acupoint (109)	The peppermint oil hot towel helps reduce abdominal distension and abdominal pain, while moxibustion with ginger at the Shenque (RN8) acupoint can alleviate symptoms of intestinal obstruction	Enhances the patient’s bowel sounds, promotes bowel movements and gas elimination, and alleviates symptoms such as abdominal pain	95.45%
External application of Chinese medicine combined with enema treatment	Manganese applied externally to the umbilicus, along with herbal enemas	Along with gastrointestinal decompression, rehydration, and other treatments, 250 g of *manganese nitrate* is placed in a cotton bag, which is sealed and laid flat on the umbilicus, then fixed in place and replaced every 12 h. Additionally, *Major Purgative Decoction* is decocted into 100 mL of medicinal liquid, which is administered as an enema twice a day (110)	The Chinese medicine enema allows the drug to be absorbed through the mucous membrane of the rectum, enhancing the bioavailability of the medication	The therapy alleviates abdominal pain and bloating while promoting anal defecation and the elimination of gas	/

## Mechanism of TCM in the treatment of AIO

Unlike barrier therapies and antibiotic treatments, which carry risks such as secondary infections and antibiotic resistance, traditional herbal medicine offers the advantage of targeting multiple pathways with fewer side effects. TCM is increasingly recognized for its role in health maintenance and disease treatment. Specifically, it addresses intestinal obstruction by enhancing gastrointestinal motility, promoting tissue repair, reducing inflammation and exudation, improving intestinal circulation, and ultimately aiding in the recovery of intestinal function. Additionally, TCM components alleviate pain, reduce gastrointestinal bloating, promote motility, and restore intestinal patency. They also regulate metabolic pathways, modulate gut microbiota, and protect mucosal integrity, further contributing to the management of intestinal obstruction. Furthermore, acupuncture plays a role in regulating the vagus nerve to stimulate gastrointestinal activity.

### Promotes gastrointestinal motility

TCM formulations exert diverse mechanisms of action in treating AIO. For instance, *Major Purgative Decoction* enhances plasma gastric motility and promotes gastrointestinal peristalsis (56). *Rhei radix et rhizome* increases colon water content, facilitating the movement of colonic contents (57). *Citrus aurantium L*. regulates gastrointestinal function through the 5-hydroxytryptamine (5-HT) signaling pathway. Active ingredients in *Kuanchangshu granules*—a formula containing *Rhubarb* (root), *Raphani Semen* (seed), and *Aurantii Fructus* (fruit)—regulate the AKT/HSP90AA1/eNOS pathway, repair intestinal tissue, promote peristalsis, and inhibit catalase secretion in the distal ileum (58). TCM also modulates gastrointestinal hormones: studies show that certain active ingredients increase ghrelin concentrations and receptor expression while inhibiting obestatin and its receptor. These effects, alongside increased secretion of gastrointestinal hormones such as Motilin (MTL) and Vasoactive Intestinal Peptide (VIP), improve gastrointestinal transmission and are beneficial in postoperative intestinal obstruction (59). Ginger-insulated moxibustion—placing a fresh ginger slice between the moxa cone and the skin—has been shown to adjust gastrointestinal motility by decreasing 5-HT receptor levels. This method also promotes inflammatory healing and intestinal mucosal repair, demonstrating particularly effective therapeutic outcomes for cancer-related incomplete intestinal obstruction (60).

### Reduce inflammatory response

TCM can prevent intra-abdominal adhesions, with its mechanism of action likely involving the inhibition of inflammation and fibrosis combined with neovascularization during adhesion formation (61). *Changtong Oral Liquid*, a TCM formula consisting of *Aurantii Fructus* Imm*a*turus (fruit), *Rhubarb* (root), and *Salviae Miltiorrhizae Radix et Rhizoma* (root), has been shown to reduce serum levels of inflammatory factors such as TNF-α, Interleukin-1β (IL-1β), transforming growth factor-β (TGF-β), and Interleukin-6 (IL-6), thereby preventing the formation of intestinal adhesions in model rats (62). A web-based pharmacological study of the TCM formula *Rhubarb and Aconite Decoction*, comprising *Rhubarb* (root, 9 g), *Aconiti Lateralis Radix Praeparata* (root, 12 g), and *Asari Radix* et *Rhizoma* (root, 3 g), suggests that it may improve AIO treatment by modulating signaling pathways such as PI3K/AKT, HIF-1, and by reducing the expression of inflammatory factors like TNF-α, IL-6, inducible nitric oxide synthase (iNOS), and cyclooxygenase-2 (COX-2) (63). *Major Purgative Decoction*, a classic TCM formula, improves the pathological damage of ileocecal intestinal tissues in a rat model of intestinal obstruction, reduces inflammation, regulates the dysregulation of nucleotide-binding oligomerization domain receptor protein (NLRP3), apoptosis-associated speckled protein (ASC), and cystathionine peptidase-1 (Caspase-1), and modulates serum levels of IL-1α, IL-1β, and IL-6, thereby alleviating the symptoms of intestinal obstruction in the rat model (64). *Emodin*, an extract from TCM, prevents the reduction of gastrointestinal motility in rats with postoperative intestinal obstruction by inhibiting the VIP/cAMP/PKA signaling pathway. It also reduces the expression of inflammatory factors TNF-α and IL-1β and increases the expression of superoxide dismutase (SOD) and glutathione peroxidase (GSH-Px) while decreasing malondialdehyde (MDA) levels, playing a therapeutic role in recovery (65).

### Regulation of metabolic pathways

TCM can also prevent and treat intestinal obstruction by regulating metabolic pathways. Studies indicate that the *Four Gentlemen Decoction*, a TCM formula comprising *Ginseng Radix et Rhizoma* (root, 9 g), *Atractylodis Macrocephalae Rhizoma* (root, 9 g), *Poria* (sclerotium, 9 g), and *Glycyrrhizae Radix et Rhizoma* (root, 6 g), promotes intestinal function recovery and regulates systemic immunity and nutritional balance in rats with an intestinal obstruction model by modulating the arginine metabolic pathway and phospholipid metabolic pathway (66).

### Regulates intestinal flora

Furthermore, TCM can address intestinal obstruction by regulating intestinal flora. The *qi*-tonifying and *blood*-circulating method, a TCM approach, has been found to reduce *E. coli* and *enterococci* counts while increasing *Bifidobacterium* and *Lactobacillus* populations, thus modulating the intestinal flora distribution in patients and aiding in the treatment of intestinal obstruction (67).

### Protects intestinal mucosal integrity

In TCM acupoint therapy, *Tongshu Gao*, composed of *Millipede* (whole millipede), *Strychni Semen* (seed), *Momordicae Semen* (seed), *Aconiti Radix* (root), *Aconiti Kusnezoffii Radix* (root), *Olibanum* (resin), and *Myrrha* (resin), is applied to the Guanyuan (RN4) acupoint. Combined with Moxibustion therapy, which involves the use of burning *mugwort* (moxa) or other substances near or on specific body areas to relax, warm, and tonify, this therapy helps move *qi* through the internal organs. It also protects the integrity of the intestinal mucosa by scavenging oxygen radicals, antagonizing lipid peroxidation, and reducing the extent of intestinal tissue damage in a mouse model of incomplete intestinal obstruction (68).

### Modulates the vagus nerve

Acupuncture also regulates various internal organ functions by modulating the vagus nerve, which in turn affects gastrointestinal activity (69). Studies have shown that acupuncture stimulates vagal and parasympathetic pathways, promoting intestinal peristalsis and facilitating postoperative recovery by preventing ileal mucosal injury through autonomous mechanisms (70). Additionally, acupuncture enhances gastrointestinal activity by repairing the ultrastructure of Cajal mesenchymal cells and restoring their function (71). Acupuncture exhibits anti-inflammatory effects during the early stages of intra-abdominal adhesion formation, promoting gastrointestinal function by reducing immunoreactivity in the gastrointestinal tract, alleviating inflammation, improving blood circulation, and limiting angiogenesis, thus reducing adhesion occurrence (72). Common acupoints used to treat AIO include Tianshu (ST25), Shuidao (ST28), Zusanli (ST36), Shangjuxu (ST37), and Zhongwan (RN12).

From the perspective of TCM, AIO is believed to stem from a deficiency of *qi* and *blood*, which impedes smooth circulation and prevents the excretion of metabolic waste from the gastrointestinal tract. Another view posits that the body accumulates *dampness*, *heat*, and *blood* stasis, obstructing the flow of *qi* and *blood*, thereby leading to AIO. Acupuncture helps promote *qi* and *blood* circulation and restores normal gastrointestinal function. However, the efficacy of acupuncture depends on the selection of acupoints and techniques. Further research is needed to identify fixed, effective acupuncture sites, and large-sample, multicenter clinical studies are required to assess its clinical effectiveness. Studies suggest that AIO is mediated by an overproduction of cytokines in the intestine, which interferes with gastrointestinal motility. Cholinergic nerves regulate cytokine responses and inhibit inflammation; therefore, acupuncture stimulation of the vagus nerve may enhance motility and modulate cytokine production. Acupuncture’s impact on vagal activity provides a scientific basis for its use in treating AIO (73).

TCM can be combined with acupuncture, moxibustion, and modern chemical drugs to enhance treatment outcomes. Recent clinical trials examining herbal medicines have shown that patients treated with these remedies have better recovery rates and no serious complications. With its rich diversity of resources and multi-target synergistic effects, TCM can promote gastrointestinal motility from various directions and angles, offering significant potential in the treatment of AIO (74). However, research exploring the precise mechanisms of action of TCM in treating AIO remains insufficient (75). While the detailed mechanisms are yet to be fully explored and verified, more practitioners and researchers are delving deeper into the drugs and techniques used, and it is anticipated that the mechanisms of TCM in AIO treatment will be gradually uncovered in the near future.

## Discussion

TCM is a valuable adjunct in treatment, with its distinct advantages when used alongside other therapies. Over its 2,000–3,000 years of history, TCM has developed a unique diagnostic and therapeutic system, primarily utilizing natural products. These are prepared through decoction or grinding to extract their active ingredients for disease prevention and treatment. Several herbs can be used alone or in combination, tailored to the specific symptoms of the patient, allowing for individualized treatments (76). TCM operates under the belief that disease results from imbalances and disharmony between the body’s organ systems and the environment. Thus, its goal is to restore harmony and balance between the body and its surroundings (77).

In TCM, intestinal obstruction is referred to as “Chang Jie” and “Guan Ge.” The main causes of this condition are thought to stem from a weak body, fragile stomach and intestines, and insufficient transformation of *qi* and *blood*, which hinders the excretion of metabolic wastes. Another contributing factor is the presence of heat, cold, dampness, *blood* stasis, and other pathogenic factors obstructing the flow of *qi* and *blood*. According to TCM, the primary pathogenesis of intestinal obstruction involves dysfunction of the internal organs and *qi*, representing an emergency within the six internal organs. Therefore, the treatment of intestinal obstruction should focus on unblocking the intestinal tract, promoting lower digestive function, warming the *qi*, dispelling cold, and alleviating pain.

TCM attributes AIO to factors such as dietary imbalances, the invasion of cold and dampness, surgical damage to *qi*, and emotional stress. The condition primarily manifests as abdominal pain, distension, vomiting, and constipation. In clinical scenarios where surgical procedures may lead to adhesion formation, TCM presents a conservative yet advantageous alternative. TCM enhances intestinal peristalsis and alleviates obstruction symptoms through the use of herbal medicine, moxibustion, acupuncture, and other therapeutic methods (78). By inhibiting inflammatory responses, TCM prevents abdominal adhesions in animal models (79), whereas postoperative inhibition of gastrointestinal motility exacerbates adhesions; thus, promoting gastrointestinal motility plays a critical role in preventing the formation of postoperative adhesions (80). Visceral massage immediately following surgery facilitates peristalsis in rats, mitigating postoperative adhesion formation (81). Herbal remedies like *rhubarb*, *moksha*, and *flaxseed* have demonstrated effectiveness in treating gastrointestinal motility disorders (82), and *emodin* has been recognized for its anti-inflammatory properties alongside its gastrointestinal-regulating effects (83). Components of the *Fructus Aurantii-Magnoliae Officinalis Cortex* herb pair also enhance gastrointestinal motility by modulating muscarinic and, secondarily, alpha receptors (84). TCM demonstrates significant clinical efficacy in the treatment of AIO. Specifically, Major Bupleurum Decoction has been shown to effectively mitigate inflammatory responses and reduce intraluminal pressure in AIO patients (85). The combination of *Rheum officinale* (Dahuang) and *Prunus persica* (Taoren) decoction with Zusanli (ST36) acupoint injection significantly alleviates abdominal distension and pain, while reducing the need for surgical intervention (86). Additionally, acupuncture regulates gastrointestinal motility, modulates gut microbiota, and protects and repairs the intestinal mucosal barrier (87). Furthermore, moxibustion therapy promotes intestinal gas passage, enhancing recovery in AIO patients (88). Thus, TCM effectively regulates gastrointestinal motility to improve peristalsis, offering significant therapeutic potential for treating AIO.

As a complementary therapeutic approach, TCM offers a safe and effective method for the treatment and prevention of AIO. TCM can combat abnormal infections, promote tissue repair, reduce inflammation and exudation, improve intestinal circulation, and ultimately facilitate the recovery of intestinal function (61). Unlike conventional treatments that typically target a single pathway, TCM works through multiple regulatory pathways, which synergistically produce pharmacological effects (21). *Major Purgative Decoction*, a classical remedy recorded in the *Shanghan Lun*, was originally prescribed for intestinal obstruction in the *Yangming meridian* and remains one of the most widely used TCM formulae for treating intestinal obstruction today. Studies indicate that *Major Purgative Decoction* effectively alleviates persistent abdominal distension and pain associated with intestinal obstruction (39). Furthermore, the decoction regulates the levels of small intestinal epithelial tight junction proteins (Claudin) in rat intestinal mucosa, even after the obstruction resolves, thus improving intestinal mechanical barrier function in rats with an intestinal obstruction model (89). According to TCM principles, acupuncture enhances the circulation of *qi* and *blood*, promoting the elimination of cold, dampness, and stagnant blood, ultimately restoring intestinal function. Moxibustion, based on TCM theory, warms the *yang qi* to pass through the meridians, dispersing cold and alleviating pain. It is effective in unblocking the meridians and promoting intestinal motility (90). Additionally, Chinese medicine retention enemas allow direct absorption of the medicinal compounds through the intestinal mucosa, shortening the drug’s onset time. This method promotes gastrointestinal smooth muscle peristalsis, enhances intra-abdominal blood flow, and improves intestinal wall circulation, while also exhibiting antibacterial and anti-inflammatory effects (91).

Although TCM offers a wide range of methods and a diverse array of remedies for treating AIO, its complexity presents challenges in fully elucidating the precise mechanisms of action. Additionally, the nature of TCM, which involves decocting herbs for patient use, makes it difficult to ensure consistent and accurate drug dosages. This variability has led some scholars to question the safety of TCM. The preparation of herbal decoctions often involves a combination of various herbs, and due to technical deviations by practitioners, the composition of these preparations may lack uniformity. While the increasing use of TCM granules is helping to address this issue, traditional decoction methods remain the dominant practice. As a result, standardizing the drugs and methods used in TCM for treating AIO requires further refinement. Nevertheless, TCM may induce certain adverse effects, predominantly drug hypersensitivity reactions. The most frequent clinical manifestations include cutaneous symptoms (erythematous rashes, pruritus, and maculopapular eruptions) and gastrointestinal disturbances (vomiting, diarrhea, and abdominal pain). Topical TCM applications also possess sensitization potential, typically manifesting as contact dermatitis and vesicular eruptions, which are frequently misidentified as simple irritant reactions. These allergic responses correlate not only with individual patient predisposition but also with specific herbal constituents. Consequently, clinical practice necessitates stricter standardization of TCM production and processing protocols, enhanced quality control measures with rigorous compositional analysis, and the implementation of evidence-based prescribing guidelines to minimize adverse events stemming from variable herbal composition or substandard product quality (86). Currently, TCM standardization is progressing rapidly, supported by the Chinese government. This process is being implemented across multiple levels, including medicinal materials, herbal pieces, TCM formula granules, herbal extracts, and proprietary Chinese medicines. Such efforts are essential for optimizing and advancing TCM. Given the complexity of TCM compositions, maximizing the extraction of active ingredients from herbs, minimizing the waste of residual materials, developing secondary products, and refining extraction techniques will all contribute to the standardization process. The multi-component, multi-target approach of TCM poses challenges in synthesizing single components akin to modern pharmaceuticals. However, this characteristic allows TCM to exert therapeutic effects in diverse ways, with certain herbs even demonstrating bidirectional regulatory effects similar to probiotics. While the mechanisms of TCM remain incompletely understood, its multi-component nature may contribute to fewer side effects compared to single-component modern synthetic drugs, which are often associated with adverse reactions. This potential for fewer side effects is one reason for TCM’s broad public acceptance. External TCM therapies also require further standardization. Although acupuncture has achieved a high level of standardization and is widely recognized by international medical communities, other external treatments, such as herbal compresses, herbal enemas, and acupoint applications, still require more precise standardization in terms of dosages and treatment protocols.

The efficacy of TCM can vary significantly due to individual patient differences, as well as the experience of practitioners in drug formulation, dosage, timing, and treatment duration. These factors contribute to inconsistent therapeutic outcomes. Despite a large user base, TCM still lacks sufficient large-scale, multi-center, evidence-based studies to verify its safety and efficacy. To address these limitations, future efforts should focus on standardizing the dosage of Chinese medicine, minimizing deviations in drug composition caused by preparation methods, and improving the consistency of the medicinal formulations. Additionally, specialized training for TCM practitioners is essential to enhance their technical expertise. Further research into the mechanisms of action of TCM in treating AIO is necessary, alongside large-sample, multi-center, evidence-based studies, which remain the most reliable means to substantiate the safety and effectiveness of TCM.

TCM treatment emphasizes individualized care, yet ensuring consistency and reproducibility of treatment outcomes presents a significant challenge in clinical practice. While personalized treatment can better address the unique needs of patients, it may also result in diversity and complexity in treatment plans, potentially affecting the consistency of clinical results. To overcome this challenge, TCM researchers have proposed various strategies in recent years, including standardizing diagnostic procedures, developing evidence-based treatment guidelines, and leveraging modern technologies such as artificial intelligence and big data analysis to optimize treatment approaches. For instance, establishing standardized TCM diagnostic criteria and treatment protocols can help reduce variations in treatment practices among practitioners. Furthermore, systematically integrating TCM with modern medicine provides a promising direction for AIO treatment, significantly improving overall therapeutic outcomes. The combination of modern pharmacology and clinical research to create evidence-based TCM guidelines also enhances the reproducibility and consistency of treatments. In the early stages of AIO, TCM therapies—such as herbal formulations and acupuncture—can be combined with conventional Western treatments like gastrointestinal decompression and nutritional support to alleviate symptoms and promote intestinal recovery. For postoperative patients, TCM methods (such as herbal medicines for replenishing *qi* and activating *blood* circulation) can complement Western rehabilitation therapies to reduce the risk of recurrence and expedite recovery. Additionally, modern medical diagnostic techniques, including imaging and laboratory tests, provide more precise evidence for TCM syndrome differentiation and treatment, thereby optimizing therapeutic plans.

Although TCM shows promise in AIO treatment, it is not universally applicable. Existing research suggests that TCM is particularly effective in the early stages of AIO (such as incomplete intestinal obstruction) and in preventing postoperative recurrence. Herbal formulations have been shown to alleviate early-stage symptoms by promoting intestinal motility and reducing inflammation. However, for cases involving acute complete intestinal obstruction or severe complications, surgery remains the preferred approach, with postoperative TCM treatment aiding recovery. Therefore, future research and clinical practice must further define the specific application of TCM across different stages and types of AIO to ensure the safety and efficacy of treatments.

Given the high incidence of AIO, recurrence rates, and the associated medical and economic burdens, preventing and minimizing postoperative adhesions is a top priority. Any preventive strategy must be safe, practical, and cost-effective (12). The primary approach to limiting morbidity and reducing adhesion-related complications is to prevent the formation of postoperative adhesions. Current preventive measures include modifications to surgical techniques to minimize tissue damage and the use of physical barriers (15). Surgeons should adhere to surgical principles to avoid excessive peritoneal dissection and spillage of bowel contents (92). Choosing the appropriate surgical technique is crucial to preventing adhesion formation. Gentle tissue handling and meticulous dissection are essential for minimizing tissue damage and inflammation and preventing plasma membrane injury (93). Additionally, minimizing the exposure and drying of the intestinal surface, as well as removing residual tissue fragments, can reduce the risk of adhesion formation (94). The use of foreign materials, such as talc, cotton wool, and fibrinogenous suture materials, should be avoided (95). Factors related to the surgical environment, such as air handlers and powder-free gloves, can also help reduce the chances of peritoneal adhesion formation. Preventive strategies also include a combination of various therapeutic approaches, surgical techniques, mechanical barriers, chemicals, and TCM interventions. However, there remains a need for more effective and safer therapeutic measures to prevent adhesions, improve treatment outcomes, and reduce recurrence rates.

## Conclusion

Although TCM shows considerable therapeutic potential in both treatment and prevention of AIO, its pharmacological mechanisms remain insufficiently elucidated. To establish robust clinical evidence, large-scale, multicenter randomized controlled trials are required to systematically evaluate TCM’s safety and efficacy profile. Additionally, well-designed experimental studies are needed to comprehensively assess the therapeutic value and safety parameters of TCM formulations, herbal medicines, and associated treatment modalities.
